# Human Induced Lung Organoids: A Promising Tool for Cystic Fibrosis Drug Screening

**DOI:** 10.3390/ijms26020437

**Published:** 2025-01-07

**Authors:** Anna Demchenko, Maxim Balyasin, Aleksandra Nazarova, Olga Grigorieva, Irina Panchuk, Ekaterina Kondrateva, Vyacheslav Tabakov, Olga Schagina, Elena Amelina, Svetlana Smirnikhina

**Affiliations:** 1 Laboratory of Genome Editing, Research Centre for Medical Genetics, Moskvorechye, 1, 115522 Moscow, Russia; demchenkoanna@med-gen.ru (A.D.);; 2 Scientific and Educational Resource Center, Peoples’ Friendship University of Russia, Miklukho-Maklaya, 6, 117198 Moscow, Russia; 3 Department of Cell Technology, Endocrinology Research Center, Dm. Ulyanova Str., 11, 117292 Moscow, Russia; 4 Moscow Branch of the Biobank “All-Russian Collection of Biological Samples of Hereditary Diseases”, Research Centre for Medical Genetics, Moskvorechye, 1, 115522 Moscow, Russia; 5 DNA-Diagnostics Laboratory, Research Centre for Medical Genetics, Moskvorechye, 1, 115522 Moscow, Russia; 6 Laboratory of Cystic Fibrosis, Research Institute of Pulmonology, 11th Parkovaya Str., 32/4, 105077 Moscow, Russia

**Keywords:** cystic fibrosis, CFTR modulators, forskolin-induced swelling assay, human induced pluripotent stem cells, lung organoids, airway basal cells

## Abstract

Cystic fibrosis (CF) is an autosomal recessive disorder caused by mutations in the *CFTR* gene. Currently, CFTR modulators are the most effective treatment for CF; however, they may not be suitable for all patients. A representative and convenient *in vitro* model is needed to screen therapeutic agents under development. This study, on the most common mutation, F508del, investigates the efficacy of human induced pluripotent stem cell-derived lung organoids (hiLOs) from NKX2.1+ lung progenitors and airway basal cells (hiBCs) as a 3D model for CFTR modulator response assessment by a forskolin-induced swelling assay. Weak swelling was observed for hiLOs from NKX2.1+ lung progenitors and hiBCs in response to modulators VX-770/VX-809 and VX-770/VX-661, whereas the VX-770/VX-661/VX-445 combination resulted in the highest swelling response, indicating superior CFTR function restoration. The ROC analysis of the FIS assay results revealed an optimal cutoff of 1.21, with 65.9% sensitivity and 71.8% specificity, and the predictive accuracy of the model was 76.4%. In addition, this study compared the response of hiLOs with the clinical response of patients to therapy and showed similar drug response dynamics. Thus, hiLOs can effectively model the CF pathology and predict patients’ specific response to modulators.

## 1. Introduction

Cystic fibrosis (CF) is an autosomal recessive disease caused by pathogenic variants in the cystic fibrosis transmembrane conductance regulator (*CFTR*) gene [1]. To date, the international CFTR2 project has described 719 pathogenic genetic variants in the CFTR gene that lead to CF, with the F508del mutation being the most common (https://cftr2.org, accessed on 5 December 2024). The F508del mutation led to impaired folding and transport of the CFTR protein to the apical membrane of epithelial cells, resulting in a marked deficiency of ion channels that are encoded by the *CFTR* gene [2]. The current treatment for CF consists of symptomatic and pathogenetic therapy. Symptomatic therapy is aimed at reducing the symptoms of the disease and includes anti-inflammatory and antibacterial therapy, enzyme replacement therapy, bronchiectasis therapy, etc. [3]. Pathogenetic therapy is aimed at correcting the structural abnormalities of the CFTR protein and/or restoring CFTR channel conductance, which is achieved through the use of modulators. However, patients carrying rare mutations do not receive therapy because there are no CFTR modulators approved for their genotypes on the basis of clinical trials [4].

Cellular models (immortalized cell lines, primary human bronchial epithelial cells and intestinal organoids) that model CF are used for *in vitro* evaluation of the efficacy of compounds for the pathogenetic treatment of CF [5,6,7]. Since the respiratory system accounts for the majority of lesions in CF [8], lung cell cultures may be an ideal model for the study of CF, as well as for developing and evaluating the effectiveness of therapies. Among lung cellular models, 3D cell cultures (organoids) best model the structural, phenotypic and functional characteristics of the native organ. The generation of primary cells for the formation of lung organoids is accompanied by highly invasive procedures. Consequently, a promising direction is to obtain human induced pluripotent stem cells (hiPSCs), which are generated by reprogramming skin cells, blood cells, urine and hair follicles [9,10], with subsequent differentiation in the epithelial direction. Biological materials for the production of hiPSCs are confirmed to be affected by CF, so they may be obtained to generate hiPSC-derived LOs from CF patients.

The efficacy of CF pathogenetic therapy can be assessed *in vitro* by measuring the functional activity of the CFTR channel. One common method is forskolin-induced swelling (FIS) of organoids/spheroids [11]. When compared with that of organoids from healthy donors, the swelling of organoids obtained from CF patients is significantly reduced or absent [12]. To date, the most common model is the intestinal organoid model. According to published clinical trials [7,13], the restoration of CFTR channel function using CFTR modulators, as assessed by FIS, correlates with improvements in lung function and the sweat chloride concentration (SCC). However, there are clear physiological differences between the respiratory mucosal surface and the gut [14,15]. Consequently, there is a question as to the representativeness of the gut epithelium in comparison to it is respiratory counterpart [16].

The generation of airway organoids (AOs) from primary cell cultures for estimation of CFTR channel function has been described [17,18,19]. For example, the response to forskolin in CF organoids was found to be lower than that detected in wild-type organoids. This correlation was consistent with the severity of the CFTR genotypes. Additionally, the addition of the CFTR modulators VX-770 and VX-809 resulted in increased swelling, which is in accordance with clinical data [18]. The response of AOs derived from hiPSCs to CFTR channel modulators for CFTR class 1–3 mutations was also evaluated [20,21]. Additionally, the potential use of therapeutic compounds to correct the CFTR protein in class 1 mutations has been demonstrated [22]. However, AOs are not very convenient cellular models because they do not tolerate cryopreservation, and long-term differentiation of hiPSCs is necessary for each experiment [23,24]. Therefore, in this study, we propose the use of hiPSC-derived lung organoids (hiLOs) to assess CFTR channel function after exposure to modulators by FIS. We previously described a protocol for obtaining and characterizing hiLOs from hiPSC-derived NKX2.1+ lung progenitors and hiBCs and confirmed that hiLOs contain functional epithelial cells that may be used to assess CFTR channel activity [23,25].

The objective of the present study was to explore the possibility of applying patient-specific hiLOs from hiPSC-derived NKX2.1+ lung progenitors and hiBCs to assess the effectiveness of pathogenetic therapy for cystic fibrosis and evaluate the accuracy of the FIS assay for hiLOs by carrying out ROC analysis.

## 2. Results

### 2.1. Derivation of Cell Cultures

**Figure 1:**
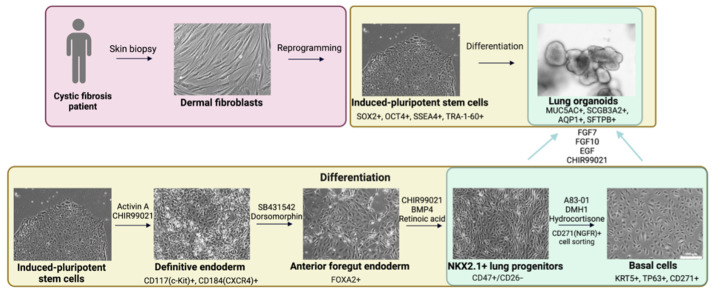
A schematic pathway for obtaining airway basal cells and lung organoids from hiPSCs. Scale bar, 100 μm. Small molecules for inducing differentiation are shown above the arrows, and cellular markers that characterize the resulting cell type are written under the images.

hiLOs generated from hiPSC-derived NKX2.1+ lung progenitors and hiBCs were considered as three-dimensional cell models for CF drug screening. hiLOs contain functional epithelial cells, which make it possible to assess the functional activity of the CFTR channel. The full scheme of the differentiation is shown in Figure 1. Complete information on the derivation and characterization of hiLOs has previously been published in our articles [23,25].

### 2.2. FIS Assay on Non-CF hiLOs

To perform the FIS assay, it was first necessary to determine the kinetics of CFTR-dependent swelling in response to forskolin in hiLOs with wild-type *CFTR*. This study was performed on two wild-type hiLO cell lines (P14L1 and P17L16) derived from NKX2.1+ lung progenitors. Two time points (5 and 24 h) and a range of forskolin concentrations (1–20 μM) were selected on the basis of previous studies [12,18,20,26,27]. For primary AOs, the incubation period with forskolin is 2–8 h, whereas for organoids derived from hiPSCs, it is 4.5–24 h [19,21,28]. The final concentration of forskolin for airway and nasal organoids ranges from 0.02 μM to 10 μM [18,19,21], and that for intestinal organoids ranges from 0.008 to 5 μM [13,27]. Since CFTR protein expression is known to be lower in airway organoids than in intestinal organoids [28], the lowest concentrations of forskolin used were not practical, so a range of forskolin concentrations from 1 to 20 μM was investigated in this study.

**Figure 2:**
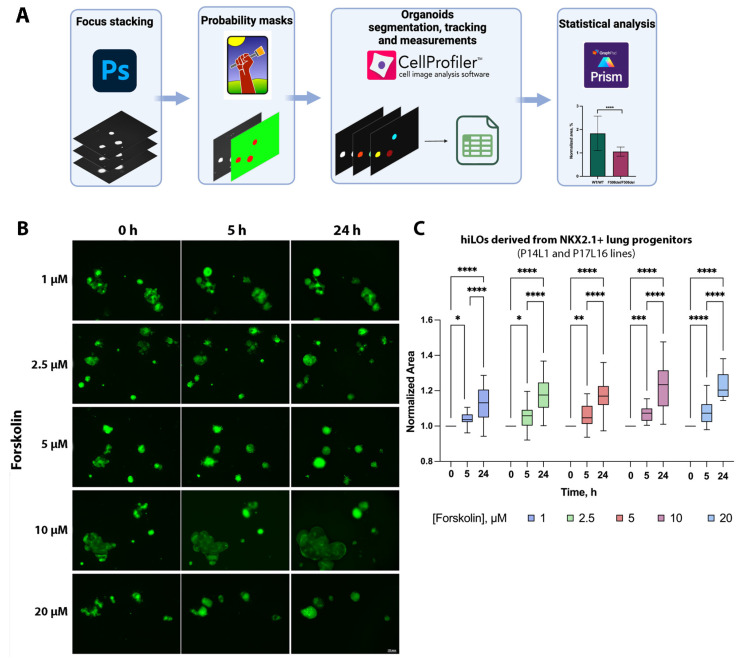
Forskolin-induced swelling of hiLOs from NKX2.1+ lung progenitors from a healthy donor. (**A**) A schematic representation of the image analysis of the FIS assay. The analysis comprises the following steps: z-stacking of organoids in different planes in Photoshop (Ps), then compilation of probabilistic masks to determine the area of the organoid in ilastik software, then measurement of the organoid area and the relation of objects over time in CellProfiler software and finally statistical analyses using GraphPad Prism. (**B**) Fluorescence microscopic images of hiLOs (P17L16 cell line) at three time points (0, 5 and 24 h) under the influence of different concentrations of forskolin. Scale bar, 100 μm. (**C**) Quantification of the normalized swelling area of the hiLOs (P14L1 and P17L16 cell lines) at 0, 5 and 24 h, calculated from the area at 0 h. The data are presented as the median, Q1-Q3, with *n* = 9 technical replicates, and the mean number of organoids analyzed in each group was 63 individual organoids. * *p* < 0.05, ** *p* < 0.01, *** *p* < 0.001, **** *p* < 0.0001.

The processing scheme of the obtained fluorescence images is shown in the schematic diagram of Figure 2A. The algorithm comprises the following steps: z-stacking of organoids in different planes; compilation of probabilistic masks to determine the area of the organoid; measurement of the organoid area and the relation of objects over time; and statistical analyses. The results of the experiment optimizing the conditions for the analysis of FIS indicated that the optimal incubation time for hiLOs with forskolin was 24 h, with an optimal final forskolin concentration of 10 μM (Figure 2B,C). This resulted in a statistically significant (*p* = 0.0467) change in the organoid area when compared with that of the control (0 h). The response (organoid area increase) was found to be statistically significantly dependent on the factor time (*p* < 0.0001 for the linear coefficient) and on the forskolin concentration (*p* = 0.0013 and 0.0153 for the linear and quadratic coefficient functions), with no statistically significant interaction between factors (time and concentration) observed (*p* = 0.4523). We observed a dose-dependent increase in the swelling of hiLOs, whereas a slight decrease in swelling was observed at 20 μM forskolin, which may be due to the cytotoxic effect of forskolin.

### 2.3. FIS Assay with Modulators on CF hiLOs

**Figure 3:**
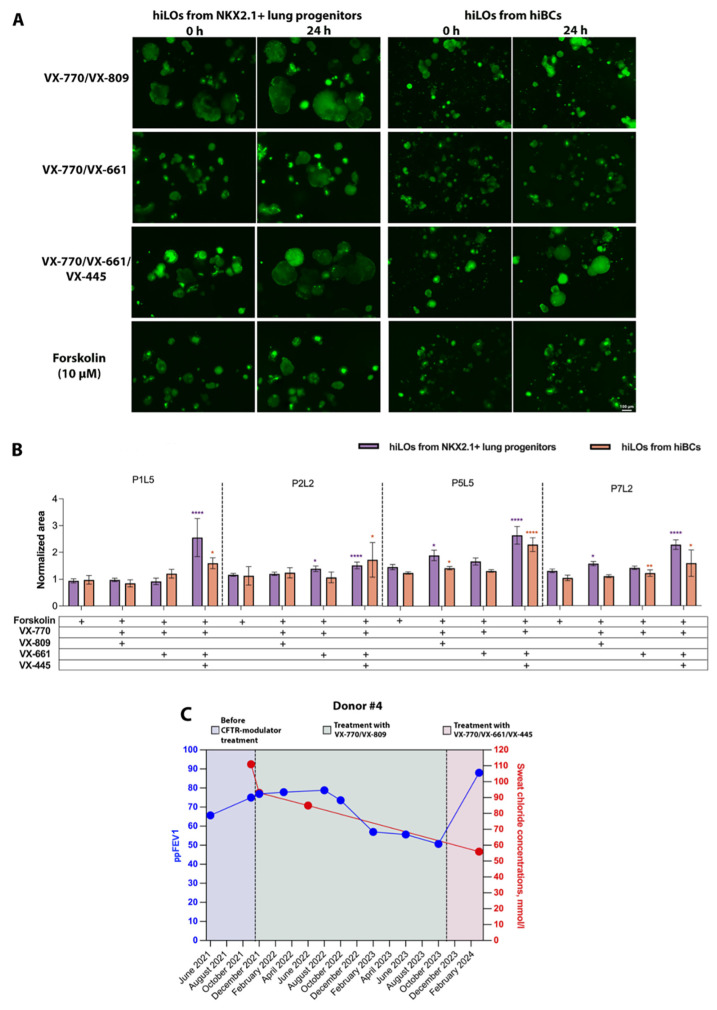
Forskolin-induced swelling of hiLOs obtained from NKX2.1+ lung progenitors and hiBCs with a homozygous F508del mutation in *CFTR* in response to CFTR modulators (mixture 1:1 of the potentiator VX-770 with the correctors VX-809, VX-661 and VX-661/VX-445). (**A**) Fluorescence microscopic images of hiLOs (P5L5 cell line) at two time points (0 and 24 h) under the influence of modulators. Scale bar, 100 μm. (**B**) Quantification of the normalized (based on time point 0) swelling area of hiLOs derived from NKX2.1+ lung progenitors and hiBCs at time points = 0 and 24 h. Data are presented as the mean ± 95% CI, *n* = 9 technical repeats, and the mean number of organoids analyzed in each group was 51 individual organoids. * *p* < 0.05, ** *p* < 0.01, **** *p* < 0.0001. (**C**) Two therapeutic endpoints (ppFEV1 (percent predicted forced expiratory volume in the first second) and sweat chloride concentration before and during CFTR modulator administration) of donor #4 with a homozygous F508del mutation in the *CFTR* before and during CFTR modulator administration.

The objective of this study was to investigate the potential of using hiLOs as cellular models to assess the correction of CFTR channel function in response to CFTR modulators. For this purpose, an FIS assay on four lines of hiLOs derived from NKX2.1+ lung progenitors and hiBCs with a homozygous F508del mutation in the CFTR (P1L5, P2L2, P5L5 and P7L2) was conducted in response to the action of three CFTR modulators (mixture 1:1 of the potentiator VX-770 with the correctors VX-809, VX-661 and VX-661/VX-445) (Figure 3A). Notably, a molecular genetic analysis for a complex allele (the combination of a homozygous F508del mutation in *CFTR* with a genetic variant p.Leu467Phe on the same allele) was previously performed in the cell lines used. The variant c.1399C>T (L467F; p.Leu467Phe) was not detected in any of the cell lines. Previous studies have demonstrated that the presence of the complex allele reduces the efficacy of CFTR modulator therapy [29,30]. When hiLOs were exposed to CFTR modulators consisting of the corrector VX-809 or VX-661 in combination with the potentiator VX-770, a weak response or no response was observed in all four hiLO lines (Figure 3B). In response to the combination of the CFTR modulator VX-770/VX-809, hiLOs derived from NKX2.1+ lung progenitors and hiBCs increased the normalized organoid area by averages of 1.56 ± 0.62-fold (SD) and 1.35 ± 0.35-fold, respectively (Supplementary Table S1). In response to the combination of the CFTR modulator VX-770/VX-661 hiLOs derived from NKX2.1+ lung progenitors and hiBCs, the normalized organoid area increased by averages of 1.47 ± 0.49-fold and 1.29 ± 0.3-fold, respectively. When exposed to triple combination therapy VX-770/VX-661/VX-445, hiLOs derived from NKX2.1+ lung progenitors and hiBCs increased the normalized organoid area by averages of 2.24 ± 1.12-fold and 1.97 ± 1.16-fold, respectively.

With one clinical example, we demonstrated that the recovery of CFTR channel function in hiLOs in response to CFTR modulators *in vitro* is similar to the patient response to modulators. Donor #4 was a woman with a diagnosis of cystic fibrosis (homozygous F508del mutation in the *CFTR*). At the time of dermal fibroblast donation, the patient’s age was 26 years. At the start of CFTR modulator therapy, the patient’s percent predicted forced expiratory volume in the first second (ppFEV1) was 75% of the proper value, and the SCC measured on the Nanoduct neonatal sweat analysis system was 111 mmol/L (Figure 3C). In the context of the CFTR modulator combination therapy VX-770/VX-809, the value of ppFEV1 increased to 76.9% of the proper value after one month but decreased to 50.7% by the end of the second year of therapy. The SCC decreased to 93 mmol/L (Nanoduct) after one month of therapy and to 85 mmol/L (Nanoduct) after six months of therapy but still remained positive (≥60 mmol/L), indicating increased sodium chloride in the sweat. The patient was subsequently prescribed a triple combination therapy with the CFTR modulators VX-770/VX-661/VX-445; after 4 months, the ppFEV1 increased to 88%, and the SCC decreased to 56 mmol/L (Macroduct (ELITechGroup, Logan, UT, USA)), which is an intermediate value (30–59 mmol/L). The *in vitro* evaluation of CFTR channel functional activity in hiLOs derived from this patient (P7L2 cell line) revealed comparable dynamics in response to CFTR modulators administered to the patient. When hiLOs derived from the patient’s NKX2.1+ lung progenitors and hiBCs were exposed to the CFTR modulator combination VX-770/VX-809, the hiLO area increased by 1.7 ± 0.4 times (*p* < 0.05) and 1.1 ± 0.1 times (*p* > 0.05), respectively. When hiLOs derived from NKX2.1+ lung progenitors and hiBCs were exposed to the CFTR modulator combination VX-770/VX-661/VX-445, the hiLO area increased by 2.3 ± 1.0-fold (*p* < 0.0001) and 1.6 ± 0.4-fold (*p* = 0.014), respectively (Figure 3B). Thus, after exposure to the VX-770/VX-809 modulator combination, hiLOs presented a slight increase in the organoid area and thus a weak recovery of CFTR channel functional activity. The patient experienced a transient increase in ppFEV1, whereas the SCC decreased but also remained elevated. However, after exposure of hiLOs to the triple combination of the modulators VX-770/VX-661/VX-445, the organoid area statistically significant increased, and the clinical parameters of the patient improved when the triple combination of the modulators almost reached normal values. On the basis of our results, we conclude that cellular models of hiLOs can probably be used to predict the clinical efficacy of CFTR modulators *in vitro*.

**Figure 4:**
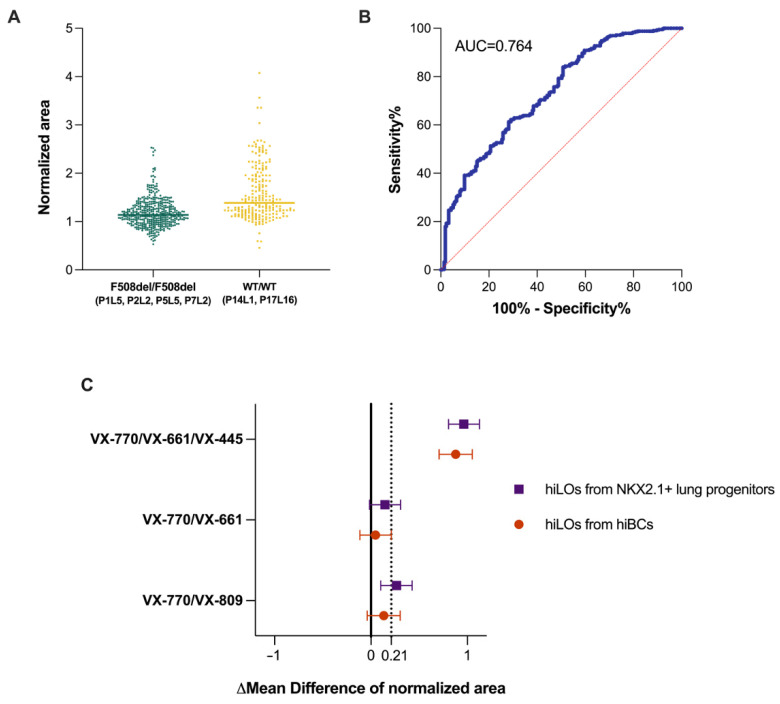
Predictive capacity analysis of hiLOs derived from NKX2.1+ lung progenitors and hiBCs from patients with a homozygous F508del mutation in the CFTR gene (P1L5, P2L2 and P7L2) and from healthy donors (P14L1 and P17L16). (**A**) Normalized area of the hiLOs after 24 h of incubation with forskolin. The mean numbers of organoids analyzed in each group were 226 for the F508del/F508del group and 125 for the WT/WT group. (**B**) ROC curve of the change in hiLO area in response to forskolin. The red dotted line represents an AUC (area under the curve) of 0.5. (**C**) The mean difference in the normalized area of hiLOs with CFTR modulators (mixture 1:1 of the potentiator VX-770 with the correctors VX-809, VX-661 and VX-661/VX-445) relative to the control group (without modulators) is represented by the mean difference ± 95% CI, GLMM. The zero line represents the statistical significance of the difference. The dotted value represents the threshold evaluated by ROC analysis.

To confirm the potential applicability of the FIS assay in the assessment of CFTR channel functional activity in hiLOs in response to CFTR modulators, the predictive efficacy of the FIS assay was evaluated. This study was conducted on four lines of hiLOs derived from NKX2.1+ lung progenitors and hiBCs with a homozygous F508del mutation in the *CFTR* gene (P1L5, P2L2, P5L5 and P7L2) and two lines of hiLOs derived from NKX2.1+ lung progenitors with wild-type CFTR (P14L1 and P17L16). The sensitivity and specificity of the method were determined using the Youden index based on ROC curve analysis. The area under the curve (AUC) was 0.764 (95% CI: 0.724–0.804), or 76.4%, with a sensitivity = 65.9%, specificity = 71.8%, cutoff = 1.21 or a 21% increase in the organoid area. This threshold was defined as the point at which the addition of forskolin resulted in a 21% increase in the organoid area when compared with the background value. An AUC ≥ 70% and <80% corresponds to a good prognostic model [31], and a positive response to forskolin is not well-established (Figure 4A,B). The associated PPV and NPV were 57% and 79%, respectively. The non-high PPV value arises because some organoids from CF patients respond to forskolin. For example, the hiLO P5L5 cell line was observed to respond to incubation with forskolin alone, and when this patient was excluded from the ROC assay at a cutoff of 1.21, the AUC value was 80.8%, sensitivity = 73.9%, specificity = 71.8%, PPV = 71% and NPV = 74%. We analyzed delta values in response to forskolin with and without CFTR modulator treatment (Supplementary Table S1). The delta values, with a cutoff of 21% (0.21), are summarized in Figure 4C. When the threshold line value (0.21) crosses the mean difference ±95% CI, we can claim a meaningful recovery of CFTR channel function in response to modulators. Figure 4C shows significant recovery of the CFTR channel function of hiLOs derived from both sources after exposure to VX-770/VX-661/VX-445 and uncertainty recovery after exposure to VX-770/VX-809 and VX-770/VX-661. In addition, the response of hiLOs from hiBCs was weaker (by 33.8%, *p* < 0.0001) than that of hiLOs from NKX2.1+ lung progenitors. The difference in the response of hiLOs formed from NKX2.1+ lung progenitors and hiBCs may be due to the difference in the proportion of CFTR-expressing cells in the organoids.

Here, for the first time, it has been shown that hiLOs derived from NKX2.1+ lung progenitors and hiBCs can serve as a cellular model used to assess the functional activity of the CFTR channel. The advantages of hiLOs derived from NKX2.1+ lung progenitors and hiBCs are an almost limitless supply of patient-specific cells, direct connection of the respiratory tract with the cystic fibrosis pathology and thus greater relevance with respect to CF modeling. The FIS assay for hiLOs has a positive predictive value and can be considered for application in the screening of new compounds for CF therapy, as described for intestinal organoids [32].

## 3. Discussion

In this article, we describe the possibility of applying lung organoids from hiPSC-derived NKX2.1+ lung progenitors and hiBCs as a cellular model to assess the efficacy of restoring CFTR channel function in response to CFTR modulators. Previously, similar works have been carried out only on AOs derived from hiPSCs [20,27] and on organoids from primary cultures (nasal and airway organoids) [17,18]. One of the advantages of our method is the possibility for the FIS assay of applying hiLOs from hiBCs, because hiBCs are known to be progenitor cells [33] and tolerate cryopreservation well [24]. hiBCs are known to tolerate cryopreservation well [24,34]. However, there are no data on successful cell cryopreservation protocols for hiPSC-derived NKX2.1+ progenitors. Therefore, it is reasonable to create a biobank of patient-specific hiBCs for subsequent hiLO formation, to use them as a model for evaluating the effectiveness of a therapy, which reduces the costs of regular directed differentiation of hiLOs directly from hiPSCs. In addition to screening pathogenetic therapies, the FIS assay on hiLOs can be used to evaluate the efficacy of emerging etiotropic therapies, such as gene therapy.

In our work, we analyzed the responses of four lung organoid lines derived from NKX2.1+ lung progenitors and hiBCs from patients with homozygous F508del mutations in the *CFTR* gene to modulators. The cell models used were characterized, and the optimal conditions for the FIS assay for hiLOs were selected (final forskolin concentration of 10 μM and incubation time with forskolin of 24 h). In our study, we observed weak swelling in response to the combinations of the modulators VX-770/VX-809 and VX-770/VX-661. Moreover, three out of the four patient-specific hiLOs produced a statistically significant response to a triple combination of CFTR modulators containing the corrector VX-445 (VX-770/VX-661/VX-445). The combinations of VX-770/VX-661 and VX-770/VX-809 modulators have relatively small clinical effects [35,36]. VX-770/VX-661/VX-445 is more effective in restoring CFTR channel function compared to VX-770/VX-661 and VX-770/VX-809 due to the use of a combination of correctors (VX-661 and VX-445), which significantly improve the folding and trafficking of F508del *CFTR*, as well as due to synergistic action with VX-770, which activates the channel. The clinical benefit of VX-770/VX-661/VX-445 is highlighted in a review summarizing data comparing the clinical efficacy of the modulators in patients with F508del in the homozygous state, with VX-770/VX-661/VX-445 demonstrating a significant increase in ppFEV1 when administered compared to VX-770/VX-661 and VX-770/VX-809 [37]. In a study by Berical et al. on hiPSC-derived AOs obtained from three different patients with homozygous F508del mutations, organoids from only one of three patients showed a significant response to exposure to combinations of the modulators VX-770/VX-809 and VX-770/VX-661, whereas exposure to triple combinations of the modulators VX-770/VX-661/VX-445 resulted in a significant response in all three organoid cultures [20].

For the ROC analysis of the FIS assay results of wild-type organoids and those with F508del in *CFTR*, an optimal cutoff of an increase in the normalized area of 1.21 was chosen, for which the sensitivity = 65.9%, specificity = 71.8%, PPV = 57% and NPV = 79%. The predictive accuracy of the FIS assay is 0.764. An accuracy of 76.4% represents the best result achieved with the predictive method described, and while this may not seem particularly high, it is a solid outcome for the relatively simple approach presented in our study. In the study by Berkers et al., the AUC for the FIS assay on rectal organoids was greater than 0.9 for predicting responders in SCC and ppFEV1 or only SCC [38]. The associated PPV and NPV were 100% and 80%, respectively. In our study, we present two clinical cases as examples: the efficacy of modulators in patients with a homozygous F508del mutation in *CFTR* and the *in vitro* response of organoids to modulators derived from these patients. Our findings demonstrate that the restoration of CFTR channel function in hiLOs in response to modulators *in vitro* reflects the clinical response to CFTR modulators in patients. However, the sample size is limited to only two patients because pathogenetic therapy is given to a very limited number of patients, and it is difficult to find patients who have cell cultures and receive therapy. Therefore, in future studies, it is necessary to recruit a sufficient sample and evaluate the accuracy of the FIS assay to detect clinical responses.

It is widely known that for rare *CFTR* genotypes in CF patients not listed in the CFTR modulator labels, the FIS assay on intestinal organoids is utilized for the assessment of therapeutic efficacy. In several countries, this method is included in clinical guidelines for CF treatment, such as in Russia [39]. Tests on intestinal organoids have demonstrated high reproducibility and relevance [40,41]. In this study, we propose an alternative method: the use of lung organoids to assess the effectiveness of CFTR modulators. Since CFTR expression is lower in lung organoids than in intestinal organoids and reflects the actual level of CFTR expression in patient lungs, this makes lung organoids a more relevant model for personalized screening of CFTR modulators, allowing for more accurate modeling of the disease pathogenesis in the lungs [42]. Also, disorders in the respiratory tract are the main cause of deaths in CF [8], and lung organoids represent a more suitable cellular model for research aimed at treating the respiratory symptoms of CF. In addition, skin fibroblast sampling is a less invasive procedure than rectal biopsy and, in our opinion, may be the preferred method when testing in children. Among the drawbacks of testing drugs for lung organoids, one can note the greater cost and duration of the procedure (for obtaining skin fibroblasts and hiPSCs and their subsequent differentiation) when compared with those of intestinal organoids. However, if, for some reason, it is impossible to perform a rectal biopsy or if the patient already has hiPSCs, then, undoubtedly, in this case, it is advisable to test them on lung organoids. Nevertheless, although the creation of hiPSCs-derived lung organoids requires a lot of effort and time, their use provides unique opportunities for more accurate modeling of CF and research of therapeutic drugs aimed at restoring CFTR channel function. A limiting factor of our study is the testing of the method only on the most common genotype in CF F508del. According to previously published studies on airway organoids from hiPSCs or nasal and intestinal organoids from biopsy material, there is a genotype-dependent response to forskolin and modulators [18,20,43,44], but the study conditions here were the same for all genotypes, so we plan to extrapolate our methodology to rarer genotypes in future studies. We can also add that from the hiPSCs with the rare nonsense mutation W1282X in a compound heterozygous state with F508del (F508del/W1282X), which we have in our cell bank [45], we derived hiLOs and performed FIS, where, as expected, we observed a lack of response to forskolin (Figure S1). Therefore, the ability to perform FIS assays on patient-specific human induced lung organoids expands the existing knowledge base on patient-specific cellular models used for screening pathogenetic therapies for cystic fibrosis.

## 4. Materials and Methods

### 4.1. Patient Materials

This study was approved by the Ethics Committee of the Research Centre for Medical Genetics (Moscow, Russia) and conducted in accordance with the provisions of the Declaration of Helsinki of 1975. Patients and healthy donors signed informed written consent forms as anonymous participants in this study and donors of biological materials. Clinical data, including baseline SSC, lung function, age, sex, CFTR genotype and respiratory microbiology, were collected from electronic medical records, deidentified and recorded (Table 1). We did not collect data on the demographics of participants, but all included patients resided in Russia. No subjects were on CFTR modulator therapy at the time of sample acquisition. Donor #4 started modulator therapy after 4 years of skin biopsy sampling.

### 4.2. Cell Culture

Skin fibroblasts from two healthy donors and four cystic fibrosis patients with homozygous F508del mutations in the *CFTR* gene were used for reprogramming. The cell lines used in this work are described in Table 2. hiPSCs were maintained in TeSR™-E8™ (STEMCELL Technologies, Vancouver, BC, Canada) on a culture dish coated with Matrigel (Corning, New York, NY, USA). All hiPSC cell lines with CF were analyzed using multiplex ligase-dependent probe amplification followed by polymerase chain reaction and fragment analysis.

The differentiation of hiPSCs from all the cell lines was carried out in accordance with a previously published protocol [23,25]. Briefly, hiPSCs were differentiated into definitive endoderm (DE) cells using 100 ng/mL Activin A (R&D Systems, Minneapolis, MN, USA) and 5 µM CHIR99021 (Tocris, Bristol, UK). Then, DE cells were subsequently differentiated into anterior foregut endoderm (AFE) cells serum-free differentiation medium (SFDM) (SFDM: 75% IMDM (Thermo Fisher Scientific, Waltham, MA, USA), 25% Ham’s F12 (PanEco, Moscow, Russia), 100× B-27 (Thermo Fisher Scientific, Waltham, MA, USA), 200× N2 (PanEco, Moscow, Russia), 0.05% bovine serum albumin solution (Sigma Aldrich, St. Louis, MO, USA), 0.45 mM 1-thioglycerol (Sigma Aldrich, St. Louis, MO, USA), 100× GlutaMAX (Thermo Fisher Scientific, Waltham, MA, USA), 0.05 mg/mL L-ascorbic acid (Sigma Aldrich, St. Louis, MO, USA) and 100× penicillin–streptomycin (PanEco, Moscow, Russia)) with 10 μM SB431542 (Tocris, Bristol, UK) and 2 μM dorsomorphin (Tocris, Bristol, UK). The AFE cells were differentiated into NKX2.1+ lung progenitor cells in SFDM with 3 µM CHIR99021, 10 ng/mL BMP4 (R&D Systems, Minnesota, MN, USA) and 100 nM retinoic acid (Sigma Aldrich, St. Louis, MO, USA).

For derivation of hiBCs from NKX2.1+, cells were cultured on basal cell medium (BCM), which consisted of PneumaCult™-Ex Plus Medium (STEMCELL Technologies, Vancouver, BC, Canada) supplemented with 1 µM A83-01 (STEMCELL Technologies, Vancouver, BC, Canada), 1 μM DMH1 (Sigma Aldrich, St. Louis, MO, USA), 0.2 μM hydrocortisone (STEMCELL Technologies, Vancouver, BC, Canada) and 100× penicillin–streptomycin (PanEco, Moscow, Russia). Then, we conducted cell sorting on the basis of the basal cell surface marker CD271 (NGFR) (BioLegend, San Diego, CA, USA). The sorted cell suspensions were cultured in BCM.

For the derivation of hiLOs, hiBCs and NKX2.1+ lung progenitors were harvested from a culture plate and resuspended in undiluted cold Matrigel. The medium for the hiLOs (lung medium) consisted of SFDM supplemented with 10 ng/mL FGF7 (R&D Systems, Minnesota, MN, USA), 10 ng/mL FGF10, 10 ng/mL EGF (R&D Systems, Minnesota, MN, USA) and 3 µM CHIR99021.

### 4.3. FIS Assay of hiLOs

To select the optimal forskolin concentration and assay time, an experiment was performed in the TIBCO Statistica v.14.0.0.15 (TIBCO Software Inc., Palo Alto, CA, USA). FIS of the organoids derived from the hiPSC P14L1 and P17L16 lines was performed on days 7–10 after passage through Matrigel. The day before analysis, the organoids were passaged in a 96-well plate with a droplet volume of 3 μL in lung medium. On the day of the analysis, calcein green (Thermo Fisher Scientific, Waltham, MA, USA) was added to the wells containing the organoids at a final concentration of 0.25 μM, and the mixture was incubated for 40 min in an incubator. Images were acquired on a Lionheart FX automated microscope in the green channel. After that, forskolin (Sigma Aldrich, St. Louis, MO, USA) was added at 5 different final concentrations (0.1, 2.5, 5, 10 and 20 μM), the samples were incubated for two time points (5 and 24 h) and images were taken. Two time points and 5 concentrations of forskolin were selected for the experiment, and the value of the normalized organoid area was calculated as the dependent variable (response) and compared to that at 0 h. A quadratic model with a two-factor linear interaction was employed. A response surface plot was constructed to assess the optimal conditions. Analysis of variance (ANOVA) was conducted to evaluate the interaction between the swelling area and exposure to various factors (time and concentration).

FIS to assess the recovery of functional activity of the CFTR channel after exposure to CFTR modulators was performed on hiLOs generated from hiPSC and hiBC lines with homozygous F508del mutations in the *CFTR* gene (P1L5, P2L2, P5L5 and P7L2). Two to three days before analysis, the organoids were passaged into a 96-well plate with a 3 μL drop volume in lung organoid medium. One day before analysis, the CFTR correctors (VX-809, VX-661 and VX-661/VX-445 (all correctors by SelleckChem, München, Germany)) were added at a final concentration of 3.5 μM, and the control group was supplemented with DMSO in a volume relevant to the volume of the CFTR correctors. On the first day of analysis, calcein green at a final concentration of 0.25 μM was added to the wells containing the organoids, which were subsequently incubated for 40 min. Images were then acquired on a Lionheart FX automated imager in the green channel. After that, forskolin was added at a final concentration of 10 μM with CFTR correctors and a CFTR potentiator (VX-770 (SelleckChem, München, Germany)). The final concentration of the potentiator was 5 μM. After incubation for 24 h, calcein green was added, and after 40 min, images were acquired on a Lionheart FX Automated Microscope.

Image analysis was performed to assess forskolin-induced organoid swelling. The methodology for this analysis was previously described [23]. The resulting images were Z-staked for autofocus purposes in Photoshop 2022 (Adobe Systems Inc., San Jose, CA, USA), then analyzed using the ilastik software v.1.3.3. (European Molecular Biology Laboratory, Hamburg, Germany) and CellProfiler software v.4.2.1. (Broad Institute of MIT and Harvard, Cambridge, MA, USA) [49,50]. The area of the organoids was normalized, with the area of each organoid at 0 h after the addition of forskolin designated 100%.

### 4.4. Statistical Data Analysis

The statistical analysis and representation of the data were conducted using GraphPad Prism v.9.1.1 and R v.4.1.2. To test the assumptions inherent to the GLMM models and check the models, the lme4 v.1.1-27.1 and performance v.0.8 libraries were employed. Descriptive statistics were calculated as the mean values with 95% confidence intervals (CI) or standard deviations (SD). To compare the normalized swelling areas of airway organoids over time, with 0 h of incubation set as a 1-fold increase, ANOVA with post hoc Tukey’s or Sidak tests was utilized, depending on the number of comparison groups. To assess the impact of the modulator on organoid swelling without the influence of individual cell cultures, GLMM models were fitted with a random intercept (cell line), and a post hoc Tukey test was conducted. To assess the impact of the organoid type on organoid swelling without the influence of individual cell cultures or the type of modulator, GLMM models were fitted with a cross random effects model (cell line and modulator), and a post hoc Sidak test was conducted. Receiver operating characteristic (ROC) analysis was conducted to determine the optimal threshold for the response to forskolin exposure between hiLOs derived from NKX2.1+ lung progenitors and hiBCs from patients with a homozygous F508del mutation in CFTR and from healthy donors. The specificity, sensitivity, positive predictive value (PPV) and negative predictive value (NPV) at the identified threshold were calculated. The data were considered statistically significant at a *p*-value of less than 0.05.

## 5. Conclusions

To summarize, our study describes the potential application of hiLOs derived from hiPSC-derived NKX2.1+ lung progenitors and airway basal cells to assess the response to CFTR modulator therapy using an FIS assay. We selected the conditions for conducting the FIS assay, evaluated the recovery of CFTR channel activity on four hiLO lines from patients with CF after exposure to CFTR modulators and demonstrated that patient-specific lung organoids have similar response dynamics on CFTR modulators when compared with patient responses. The data indicate that hiPSC-derived lung organoids are a promising tool for cystic fibrosis drug screening.

## Figures and Tables

**Table 1:** Clinical characteristics of the cell donors.

Donor	Age (Years, Taking Clinical Characteristics)	Age (Years, Sampling Skin Biopsy)	Sex	*CFTR* Genotype	SCC, mmol/L (Method)	ppFEV1	Microbiology
1	28	27	M	F508del/ F508del	66 (Gibson)	24	SA AX MA
2	3 for SCC 26 for ppFEV1	20	M	F508del/ F508del	75 (Gibson)	58	PA AF
3	33	29	M	F508del/ F508del	82 (Nanoduct)	20.8	BC
4	26	22	F	F508del/ F508del	111 (Nanoduct)	75	SA CA KO
5	-	14	M	WT/WT	-	-	-
6	-	7	F	WT/WT	-	-	-

SCC—sweat chloride concentration, ppFEV1—percent predicted forced expiratory volume in the first second, M—male, F—female, SA, *Staphylococcus aureus*; AX, *Achromobacter xylosoxidans*; MA, *Mycobacterium abscessus*; PA, *Pseudomonas aeruginosa*; AF, *Aspergillus fumigatus*; BC, *Burkholderia cenocepacia* ST709; CA, *Candida albicans*; KO, *Klebsiella oneumoniael*.

**Table 2:** List of cell lines used in this research.

Donor	Cell Line	*CFTR* Genotype	Reference
1	P1L5	F508del/F508del	[46]
2	P2L2	F508del/F508del	https://hpscreg.eu/cell-line/RCMGi013-A, accessed on 5 December 2024
3	P5L5	F508del/F508del	[47]
4	P7L2	F508del/F508del	[48]
5	P14L1	WT/WT	https://hpscreg.eu/cell-line/RCMGi014-A, accessed on 5 December 2024
6	P17L16	WT/WT	https://hpscreg.eu/cell-line/RCMGi016-A, accessed on 5 December 2024

## Data Availability

The data presented in this study are available upon request from the corresponding author.

## Supplementary Materials

The following supporting information can be downloaded at https://www.mdpi.com/article/10.3390/ijms26020437/s1.
