# Supplementary material for: Human Induced Lung Organoids: A Promising Tool for Cystic Fibrosis Drug Screening
Source: Int J Mol Sci. 2025 Jan 7;26(2):437. doi: 10.3390/ijms26020437 (PMC11764749; doi:10.3390/ijms26020437)
Supplement: Supplementary file 1 [file ijms-26-00437-s001.zip › ijms-3383003-supplementary.pdf]

Table S1 – Change in normalized area of hiLOs after 24 h of incubation with CFTR-modulators and the mean difference in the normalized area of hiLOs with modulators relative to the control group

Cell line	Source of cells for organoids	CFTR-modulators	Change in organoid area, mean ± SD	p-value	Delta value of organoids area, mean ± 95%CI
P1L5	NKX2.1+ lung progenitors	VX-770/VX-809	0.98 ± 0.15	p = 0.831	0.03 (-0.08–0.15)
		VX-770/VX-661	0.92 ± 0.22	p = 0.969	-0.02 (-0.16–0.11)
		VX-770/VX-661/ VX-445	2.55 ± 1.29	p < 0.0001	1.61 (1.15–2.07)
	hiBCs	VX-770/VX-809	0.85 ± 0.11	p = 0.627	-0.13 (-0.44–0.18)
		VX-770/VX-661	1.35 ± 0.35	p = 0.015	0.38 (0.07–0.68)
		VX-770/VX-661/ VX-445	1.59 ± 0.64	p = 0.011	0.62 (0.12–1.11)
P2L2	NKX2.1+ lung progenitors	VX-770/VX-809	1.19 ± 0.15	p = 0.948	0.04 (-0.17–0.24)
		VX-770/VX-661	1.38 ± 0.29	p = 0.014	0.23 (0.04–0.41)
		VX-770/VX-661/ VX-445	1.51 ± 0.44	p < 0.0001	0.35 (0.18–0.52)
	hiBCs	VX-770/VX-809	1.23 ± 0.25	p = 0.998	0.02 (-0.39–0.43)
		VX-770/VX-661	1.06 ± 0.21	p = 0.752	-0.15 (-0.59–0.29)
		VX-770/VX-661/ VX-445	1.72 ± 0.77	p = 0.015	0.51 (0.08–0.93)
P5L5	NKX2.1+ lung progenitors	VX-770/VX-809	1.88 ± 0.83	p = 0.001	0.43 (0.06–0.79)
		VX-770/VX-661	1.66 ± 0.63	p = 0.161	0.21 (0.14–0.55)
		VX-770/VX-661/ VX-445	2.63 ± 1.36	p < 0.0001	1.18 (0.81–1.55)
	hiBCs	VX-770/VX-809	1.21 ± 0.36	p = 0.028	0.18 (0.02–0.34)
		VX-770/VX-661	1.3 ± 0.31	p = 0.532	0.08 (-0.08–0.23)

		VX-770/VX-661/ VX-445	2.28 ± 1.24	$p < 0.0001$	0.65 (-0.36–0.93)
P7L2	NKX2.1+ lung progenitors	VX-770/VX-809	1.66 ± 0.41	$p = 0.026$	0.27 (0.03–0.51)
		VX-770/VX-661	1.41 ± 0.32	$p = 0.531$	0.11 (-0.13–0.36)
		VX-770/VX-661/ VX-445	2.28 ± 1.00	$p < 0.0001$	0.98 (0.81–1.29)
	hiBCs	VX-770/VX-809	1.12 ± 0.08	$p = 0.408$	0.07 (-0.17–0.24)
		VX-770/VX-661	1.21 ± 0.11	$p = 0.027$	0.18 (-0.17–0.24)
		VX-770/VX-661/ VX-445	1.59 ± 1.32	$p = 0.014$	0.55 (-0.19–1.29)

FIS assay of the F508del/W1282X hiLOs

hiLOs were obtained from hiPSC-derived NKX2.1+ lung progenitors according to the protocol described in Materials and Methods in this article. hiPSCs from a patient with the F508del/W1282X mutation in the *CFTR* gene were previously obtained and registered in the hPSCreg database [46]. FIS assay was performed according to the protocol described in Materials and Methods in this article with a final forskolin concentration of 10 µM and 24 h incubation time. According to the result of FIS assay, no statistically significant response to forskolin was observed in organoids ($p=0.32$). This result indicates that the selected conditions of FIS assay on hiLOs are not only applicable to F508/F508del genotype. However, further experiments are needed to expand the variants of CFTR genotypes for FIS assay on hiLOs.

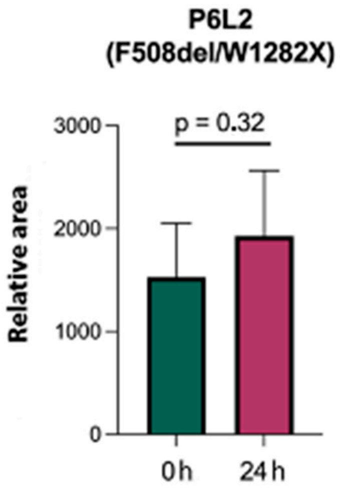

Figure S1. Forskolin-induced swelling of hiLOs from NKX2.1+ lung progenitors. Quantification of the relative area of the hiLO at 0 and 24 h. The data are presented as the mean ± SD, with n = 9 technical replicates
